# Vulnerabilities and life stressors of people presented to emergency departments with deliberate self-harm; consolidating the experiences to develop a continuum of care using a mixed-method framework

**DOI:** 10.3389/fpubh.2022.1019131

**Published:** 2023-01-11

**Authors:** Saju Madavanakadu Devassy, Lorane Scaria, Jaicob Varghese, Anuja Maria Benny, Nicole Hill, Lynette Joubert

**Affiliations:** ^1^Department of Social Work, Rajagiri College of Social Sciences (Autonomous), Kochi, Kerala, India; ^2^International Centre for Consortium Research in Social Care (ICRS), Rajagiri College of Social Sciences (Autonomous), Kochi, Kerala, India; ^3^Department of Social Work, Melbourne School of Health Sciences at the University of Melbourne, Parkville, VIC, Australia; ^4^Department of Critical Care Medicine, Rajagiri Hospital, Kochi, Kerala, India

**Keywords:** self-harm, psychosocial factors, emergency departments, India, trauma care

## Abstract

**Objective:**

Suicide is a crucial public health concern. However, the interactions between bio psychosocial vulnerabilities and stressors leading to deliberate self-harm behavior remain unexplored, especially in the Indian context. This study examined the experiences leading to self-harm behavior among people who presented to emergency departments with suicidal attempts.

**Methods:**

In this mixed-methods study, we enrolled 44 patients who presented with self-harm behavior at three tertiary health care facilities between October and December 2019. To collect quantitative data, we employed standardized tools: General Health Questionnaire (GHQ-28), General Help-Seeking Questionnaire, Mini International Neuropsychiatric Interview, and the Brief Resilience Scale. Further, we conducted semi-structured interviews to qualitatively explore participants' life experiences and other risk factors. Qualitative analyses were performed using thematic analysis and quantitative descriptive and inferential statistics were performed using STATA software.

**Results:**

The mean age of subjects were 29.8 years. The mean suicidality score for the patients was 26 (±8.7). In univariate analysis, depression and anxiety were positively associated with suicidality. While help-seeking behavior and resilience were negatively associated with suicidality. Qualitative results were centered on three major themes; life stressors, family related stressors, and social support-related vulnerabilities. The subjects' lived experiences were introduced in the backdrop of the interplay of vulnerabilities and stressors.

**Conclusion:**

The biopsychosocial vulnerabilities remain dormant until it is activated by life stressors resulting in severe self-harm behaviors. Mental health team-driven assertive engagement, positive coping, and social support interventions would help prevent reattempts in people with self-harm behaviors.

## 1. Introduction

Suicide prevention is a top priority in the international public health agenda to achieve health for all (1). Southeast Asian regions account for one-third of suicides globally and the rate of suicide is as high as 17.2 per 10,000 population (2). India has the highest suicide rate in the Southeast Asian region (3), with huge interstate variations. For instance, Kerala has a suicide rate of more than 24.3 compared with the national rate of 10.4 per 100,000 population (4). Further, approximately 10% of people who attempted suicide were reported to complete it (5), and the risk of suicide within the first 12 months after an episode of Deliberate Self-Harm (DSH) was 37.2 times higher than a general cohort (6). However, even with this high level of risk and the need for prioritized action, people with DSH are often not followed up sufficiently to prevent future attempts.

Suicide is a complex multifactorial phenomenon, that requires a patient-oriented approach rather than a passive illness-oriented approach (7). Understanding the underlying etiological factors such as epidemiological, sociological, philosophical, psychiatric, biological, and psychological (8–15) are critical to reducing the risk of repeated suicide attempts in Indian settings (16). Several studies have documented this urgent need for context-specific and individual-centered research actions toward self-harm and suicide prevention with immediate priority in developing nations such as in India (17, 18).

We used the stress and vulnerability model (19) to explain the complex interaction of vulnerability traits often determined by biological markers from brain insults and infections (20) and psychosocial stressors of early trauma and childhood adversities (21). These vulnerability traits modulate cognitive and affective processes of rejection sensitivity, perceived inadequacy (22), reactive behaviors (23), impulsivity, pessimism, lack of help-seeking behavior, and hopelessness (24). This research is an attempt to explore the complex interplay of inherited vulnerabilities, enduring mental health issues, and the trait that evolved due to deprivations and abuses over a lifetime. Assuming that the genetic predispositions coupled with psychosocial and economic risk factors lead to negative stress reactions culminating in DSH. The experiences of the survivors would help to model targeted interventions to prevent reattempts in this high-risk population.

## 2. Methods

### 2.1. Study design

In this mixed-methods study, we used structured questionnaires and semi-structured interview schedules to concurrently collect quantitative and qualitative data. We conducted the study in the emergency departments of three private tertiary care facilities, between October 2019 and November 2019, where suicide-related emergency cases are often reported. The hospitals were selected purposively from different districts of Kerala state, India, to represent a cross-section of the population.

### 2.2. Participant recruitment

We identified 58 people fulfilling the broad eligibility criteria who accessed emergency care with DSH. The current study included people who presented to the hospital emergency after non-habitual deliberate self-harm and serious suicidal attempt (SSA), which have a high lethality and a higher chance of repeated self-harm behavior (25). “Attempted suicide” is defined as a non-fatal, self-inflicted destructive act with explicit or inferred intent to die (26). We have excluded the “Parasuicidal Pause” and “Parasuicidal Gesture” to align with the standard definition of suicidal attempt (25).

#### 2.2.1. Eligibility criteria

Inclusion—Patients admitted with DSH behaviors, aged above 16 years, admitted to Emergency medical facilities within 14 days of the index attempt, providing informed consent, and with a willingness to participate were included.

Exclusion—The patients with Parasuicidal Pause and Parasuicidal Gesture and those who did not consent to the study were excluded.

**Figure 1:**
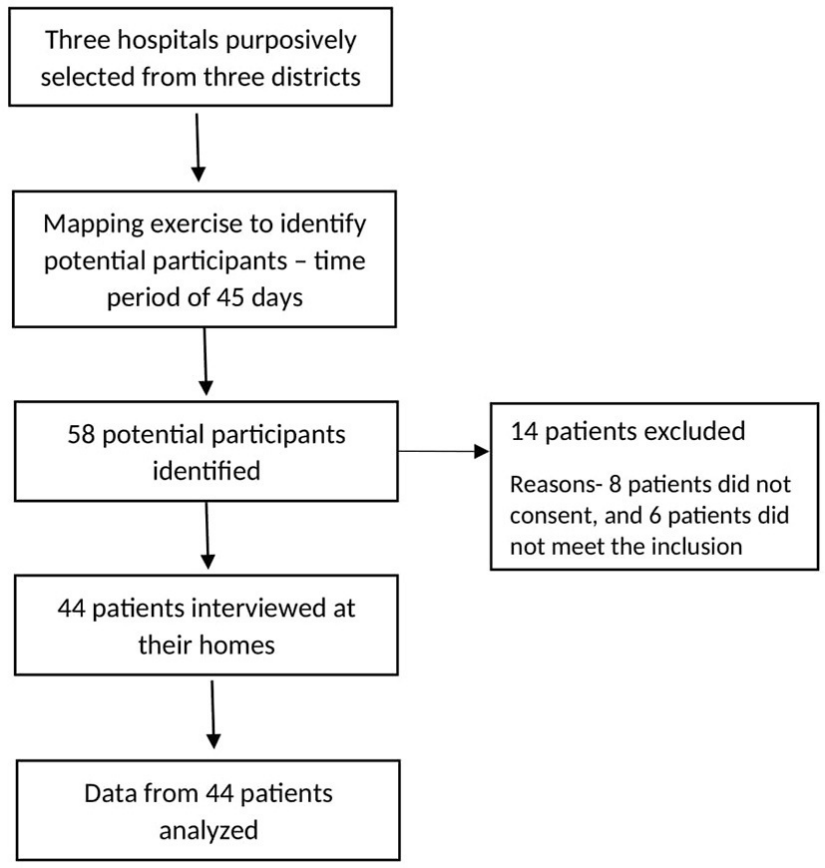
Participant recruitment from hospitals.

Out of 58 potential participants 44 were included in the study. Six of them did not fulfill the criteria and eight of them did not consent to be part of the study. The participants were recruited after obtaining written informed consent from suicide survivors and one of their immediate family members. Interviews were conducted by second-year postgraduate medical and psychiatric social work trainees (PSWTs) with mental health knowledge and research competencies. Participant data were stored in the university's secure database (password protected). Figure 1 depicts the flowchart for the recruitment of participants in this study.

### 2.3. Measurement tools

#### 2.3.1. Quantitative data collection

The following scales were used for psychosocial assessments:

Mini International Neuropsychiatric Interview (MINI): Mini International Neuropsychiatric Interview (MINI) for Suicidality Disorders Studies 7.0.2 was used to measure the level of suicidality among respondents (27). MINI is a short diagnostic tool used to assess psychiatric symptoms according to the Diagnostic and Statistical Manual (DSM) criteria. The tool is found to be reliable (Kappa coefficients ranging between 0.76 and 0.93) and valid (28). The tool has been used in Indian settings to measure psychiatric comorbidities (29).

General Health Questionnaire(GHQ-28): GHQ measures the general health of the respondents based on their responses to a set of 28 questions recorded in a four-point Likert scale ranging from “not at all,” “no more than usual,” “rather more than usual” and “much more than usual.” A higher score indicated higher level of mental health symptoms (30). The tool assesses somatic symptoms, anxiety, and insomnia, social dysfunction, and severe depression. The scale was found to be reliable (The Cronbach's alpha = 0.85) and valid (31).

General Help-Seeking Questionnaire (GHSQ): GHSQ measures the help-seeking intentions of the respondents using a set of 11 questions from both formal and informal support sources. The tool measured the help seeking behavior on a seven-point rating scale ranging from 1 to 7, 1 being highly unlikely and 7 being very likely. A higher score indicated a higher intention to seek help. The scale is found to be reliable (Cronbach's alpha =0.85) and valid (32).

Brief Resilience Scale (BRS): BRS is a 6-item questionnaire used to measure the ability to bounce back from situations (33). It is measured on a five-point scale with a higher score indicating better resilience. The scale was found to be a reliable (a = 0.71) and valid measurement tool (34).

In addition, we collected participants' demographics and social networks. The measurement tools were translated and back-translated into Malayalam (the local language) and English by two experienced researchers separately to account for validity. Each quantitative interview lasted for ~20–30 min.

#### 2.3.2. Qualitative interview

The qualitative data were collected using a literature-informed semi-structured interview guide developed by the research team. The experts finalized the open-ended and probing guide to conduct in-depth interviews after multiple deliberations. The key aspects included in the qualitative interview were the economic status of the patient/ client and family; cultural, interpersonal, and social context, community supports, situational stressors and worries, and services available and accessed. The tool included probes to obtain information regarding the family, society, support systems, economy, and workplace, which affect the psychological aspect of an individual. The researchers collected information regarding participants' concerns in physical, psychological, and social domains. The interview was conducted in the local language (Malayalam) and was audio recorded. Each interview lasted between 30 and 45 min. Recordings were transcribed verbatim and translated into English as required. Two blinded researchers coded the collected data, with an expert verifying the transcripts and codes for accuracy. Comparisons were made between the coding sets of each researcher, and areas of disagreements and discrepancies were discussed and resolved.

### 2.4. Analysis of data

Qualitative data were analyzed using an iterative process until categories and themes emerged (35). The experts from an Australian University, and an Indian University, had a series of meetings in India to have a consensus on the overarching themes and sub-themes. Statistical analyses were performed using STATA-14 (StataCorp LLC, TX 77845, USA). We used descriptive statistics to present the profile of the respondents. The regression analysis at 95% confidence intervals with *p*-values was used to determine the predictors of DSH.

### 2.5. Research team and supervision

The primary research team included one critical care physician, the medical emergency chief of the hospital, academic faculties from Rajagiri College of Social Sciences and the University of Melbourne, psychiatric social workers, and nine social work trainees. Social work trainees who undertook the data collection were systematically trained by the academicians through direct and online supervisory mechanisms. Standard operating procedures were provided and training was provided on the methods and techniques of data collection. Further, student trainees utilized mock interviews and role plays to practice the interview questions. At the hospitals, social work trainees were supervised and monitored by social workers employed at the selected hospitals where data collection took place.

### 2.6. Ethical considerations

We obtained ethical approval from the Institutional Review Board of Rajagiri College of Social Sciences with Reg No: RCSS/IEC/011/2019. Subjects were explained the involuntary nature of participation and were recruited only after obtaining informed consent from the subjects and their family members.

## 3. Results

### 3.1. Quantitative results

We included 44 participants in the study. The mean age was 29.8 (SD = 11.3) years and the majority of them were women (61.4%). Most of the participants were aged < 30 years (65.9%), and unmarried (61.4%). The prevalence of depression and anxiety was higher in employed urban participants aged above 30 years (Table 1).

**Table 1:** Characteristics of participants (*n* = 44).

**Variables**	**Frequency (%)**	Prevalence of depression^*^	Prevalence of anxiety^*^
		22.8 (4.7)	21.1 (4.4)
**Age**			
Below 30	28 (63.6%)	22.3 (4.8)	20.5 (5.1)
Above 30	16 (36.4%)	23.8 (4.7)	22.2 (2.7)
**Gender**			
Male	17 (38.6%)	24.5 (4.0)	21.6 (4.2)
Female	27 (61.4%)	21.7 (4.9)	20.8 (4.6)
**Living arrangements**			
Town	10(22.7%)	25 (3.6)	22.9 (2.7)
Village	26(59.1%)	23 (4.3)	20.8 (4.7)
City	8(18.2%)	19.5 (6.0)	19.8 (4.9)
**Marital status**			
Single	27 (61.4%)	22.7 (4.8)	21.4 (4.8)
Married	16(36.4%)	22.8 (4.8)	20.2 (3.6)
Divorced	1(2.3%)	25	27
**Employment**			
Employed	14 (31.8%)	24.4 (4.0)	22.2 (3.5)
Self employed	3 (6.8%)	24.7 (3.5)	21 (1)
Housewife/ unemployed	6 (13.6%)	20.8 (5.2)	19.5 (6.0)
Student	20 (45.5%)	22 (5.2)	20.85 (4.9)
Retired	1(2.3%)	24	21
**Education**			
Primary school	3 (0.8%)	23 (3.5)	21 (0)
Secondary school	8 (18.2%)	26.1 (2.2)	22.6 (3.8)
Higher secondary school	6 (13.6%)	22.8 (4.4)	21.2 (3.3)
Diploma certificate	4 (9.1%)	22 (4.5)	21.5 (2.1)
Undergraduate degree	18 (40.9%)	21.8 (5.5)	20.7 (5.5)
Open university	5 (11.4%)	21.6 (5.3)	20 (5.6)

^*^Depression and anxiety were measured from GHQ-28.

Table 2 lists the summative scores of significant variables of the study. The mean scores for depression and anxiety were 22.8 (SD = 4.7) and 21.1 (SD = 4.4), respectively, in the study population. 97.73% of the respondents were diagnosed with a current episode of major depression, while 77.27% had a previous history of major depression.

**Table 2:** Summative scores and frequencies of major variables.

**Variables**	**Mean (SD)**	**Median**	**Range (min–max)**	**Frequency (%)**
Depression	22.8 (±4.8)	24.5	11–28	–
Anxiety	21.1 (±4.4)	21	10–28	–
Social dysfunction	20.9 (±4.4)	21	11–28	–
Somatic symptoms	18.9 (±4.8)	19.5	10–28	–
Resilience	4.4 (±1.8)	4	2–9	–
Suicidality	26 (±8.7)	32.5	4–33	
Major depression- current	–	–	–	43 (97.7 %)
Major depression- past	–	–	–	34 (77.3%)
Generalized anxiety disorder	–	–	–	43 (97.7 %)

Table 3 shows the results of the linear regression analysis of suicidality and its associated factors. Resilience (*B* = −1.199, *p* = 0.049) and help to seek (*B* = −1.149, *p* = 0.014) were identified as protective factors and these associations remained statistically significant even after adjusting for participants' age and sex.

**Table 3:** Risk and protective factors for suicidality.

**Variables**	**Unadjusted *B* coefficients (95% CI), *p*-value**	**Adjusted *B* coefficients (95% CI), *p*-value**
**Protective factors**		
Help seeking-friends	−1.2 (−2.0 to −0.2), 0.014	−1.0 (−1.9 to −0.1), 0.034
Resilience	−1.7 (−3.1 to −0.3), 0.015	−1.71 (−3.1 to −0.3), 0.017
**Risk factors**		
Somatic symptoms	1.0 (0.6–1.5), < 0.000	1.0 (0.5–1.5), < 0.000
Anxiety	0.9 (0.4–1.5), 0.001	0.9 (0.4–1.5), 0.002
Social disapproval	0.9 (0.4–1.5), 0.001	1.0 (0.5–1.6), 0.001
Depression	1.2 (0.8–1.7), < 0.000	1.3 (0.9–1.7), < 0.000

Major risk factors associated with suicidality were somatization (*B* = 1.059, *p* < 0.000), anxiety (*B* = 0.930, *p* = 0.001), perceived social disapproval (*B* = 0.955, *p* = 0.001), and depression (*B* = 1.238, *p* < 0.000). These associations remained statistically significant even after adjusting for participants' age and sex.

### 3.2. Qualitative results

The primary focus of the qualitative inquiry was to identify the life stressors, vulnerabilities, stress related to family and social engagements that resulted in DSH in the studied population. The emerged vulnerability themes were consolidated under three overarching themes; life stressors, family-related stressors, and socio-economic related vulnerabilities (see Table 4).

**Table 4:** Qualitatively explored risk and vulnerability factors associated with suicide.

**Major theme**	**Sub-theme**	**Frequencies (%)**
*Life stressors and vulnerabilities*	Failure of romantic relationships	10 (22.7)
	Family problems	11 (25)
	Childhood neglect	12 (27.27)
	Affectionless parental control	7 (15.9)
	Loss of a loved ones	4 (9)
	Mental illness (past depression and anxiety)	34 (77.27)
	Rejection induced stress	11 (25)
	Perceived inadequacy	3 (6.8)
	Parental pressure to excel in academics	4 (9)
	Fatal chronic illness	3 (6.8)
	Other life stressors	13 (29.54)
*Stress related to family relationships and support*	Lack of protective and supportive households	28 (63.36)
	Unsupportive parents	10 (22.7)
	Abuses and violence in the family	12 (27.27)
	Marital conflicts	12 (27.27)
	Marital infidelity and marital disharmony	4 (9)
	Alcoholism-induced marital conflict and Domestic violence	8 (18.18)
*Socio-economic vulnerabilities and stressors*	Poverty and poor-income households	21 (47.72)
	Financial insecurity	18 (40.9)
	Unsupportive neighborhood	4 (9)
	Lack of support from relatives and friends	26 (59.09)
	Lack of confiding relationships	35 (79.54)
	An unsupportive and hostile work environment	9 (20.45)
	Debt	13 (29.54)
	Workplace harassment	5 (11.36)
	Job loss/unemployment	6 (13.635)
	Alcohol-induced economic issues	5 (11.36)

#### 3.2.1. Global theme 1: Life stressors and vulnerabilities

Social and interpersonal issues, including failure of romantic and family relationships, were stressors for DSH in younger participants. For instance, Mr. X mentioned, “*My girlfriend cheated on me. I sacrificed a good job and a secure life for the sake of this relationship. I could not even support my father in his economic distress” (p8, male, 23 years)*. A 22-year-old girl told*; “We were in a relationship for four years, but I realized… for him, it was only a time pass” (p27, Female 20 years); another boy said, “My girlfriend avoids me; she blocked my contacts, on WhatsApp, Instagram, and Facebook” (p23, Male, 25 years)*.

On the other hand, the stressors of married people were related to household events. A few were domestic violence, mental illness of the participant, spouse, or a family member, dowry harassment, marital infidelity, marital disharmony, and over-involvement of in-laws.

*A married woman justified her attempt by saying, “My husband beats me to near death. If I die, he will be jailed, and my children will be orphaned; if I die, my children will have at least their father to take care of them” (p28, Female, 32 years). Another participant felt stressed; “He (husband) listens only to his mother; mother-in-law instigates him against me for more dowry” (p41, Female, 24 years). Few other stressors identified include rejection-induced stress—“No one understands me even my mother” (p2, male, 20 years), loneliness—“all are selfish” (p13, male, 19 years), feeling of inadequacy—“I cannot blame anyone. I bore them… a few friends listen to me...but in fact, they just sympathize with me” (p43, male 26 years) and lukewarm attitude of the family—“they asked me to adjust and never leave this relationship” (p14, Female, 29 years)*.

A few of the additional stressors were the perceived inadequacy, “*I am an incompetent wife, that is why he is into an extramarital relationship*” (p5, Female, 31 years), and thoughtlessness, “*my father was concerned only about his (husband) family assets, and never enquired about the family pathology. He is abnormal; neglects hygiene… highly irritable… too religious... I am afraid to tell anyone about it. It may hurt others” (p33, female, 28 years)*. Pressure to perform in studies was also found stressful for a few student participants—“*My mother compelled me to do chartered accountancy, she is responsible for my sufferings” (p34, female, 21 years)*.

The suicide attempt aggravated the social stigma-induced stress, “*I am reluctant to go out... people think I am mad… the suicide attempt made my life horrible....” (p37, male, 34 years)*. Multiple stressors with narrow coping options result in DSH, “*My father is an alcoholic. He disposes of every asset to drink (Alcohol)” (p32, male, 21 years). Some consider alcohol as a self-medication to cope with life stressors “because of my nagging wife” (p17, male, 31 years), “huge debt” (p22, male, 28 years), “felt insignificant at home” (p11, male, 19 years) and “desperate due to my illness (Cancer)” (p30, male, 35 years)*. The unresolved issues, poor coping skills, and inadequate social support were associated with DSH behavior in the participants.

#### 3.2.2. Global theme 2: Stress related to family relationships and support

People who grow up in families with a deep collectivist mindset perceive family support and bonding as primary to their individual preferences. However, the failure of the family system to protect them inflicts severe stress. Ms. L stated: “*My family doesn't support me now that I've married the guy I chose. Initially, he (her husband) was very supportive. But now, he drinks (alcohol) regularly and started to suspect me of infidelity. His physical abuse is unbearable” (p39, female, 26 years). M*arital conflicts such as infidelity, domestic violence, misunderstandings, and an unsupportive family environment resulted in stress and vulnerability in a few subjects. Ms. R reported, “*My husband has an affair, and he spends most of the money on her. What will I do if he rejects me? All the assets are in his name. I have no place to go.”* (p29, female, 30 years) Another participant said, “*We were in a relationship for 8 years. But we could not withstand the objection from our families... we decided to die together” (p31, female, 19 years)*. The “*serious accidents in them or significant others” (p7, female, 22 years)*, or “*death of significant others” (p18, female, 23 years)* are vulnerability factors for SHB in a few of them. “*Recently, my brother and one of my best friends died in accidents”*. Some of the participants' narratives reveal the unconscious patriarchal influence that led to blaming a woman for a man's infidelity and reinforcing the fundamental helplessness of how a woman's life is at stake without her husband. The intricate web of psycho-socio-cultural stressors precipitates the DSH behavior where the family, if supportive, is a protective mechanism.

#### 3.2.3. Global theme 3: Socio-economic vulnerabilities and stressors

Economic factors in terms of inadequate income, financial insecurity, joblessness, and debt were chief stressors for participants. Ms. B reported, “*I'm a salesperson, and my income is spent on my family. I am being used.” (p41, male, 25 years)* Mr. S said, “*I have been an incompetent provider, but now the family is worried about my attempt. They keep an eye on me, and never allow me to go for the job” (p9, male, 31 years)*.

An unsupportive and hostile work environment was yet another determinant of DSH in early-career employees. “*My boss takes advantage of me; he harasses me to get the work done” (p43, Female, 28 years); “my superior officers never acknowledge my contribution” (p12, Male, 29 years), “I live for the bank, but they never consider me as an asset and as a hard-working person” (p14, Female, 28 years)*. Another employee felt exploited by other employees. “*They are at the finishing point to claim the credit for the work I have completed toiling day and night” (p30, Female, 31years). Another person told, “My boss takes advantage of me. My superiors are dominating and never acknowledge my work. All are using me for their advantage...I feel like I am trapped here in this office” (p20, female, 26 years)*. A newly employed felt desperate about the unsupportive *colleagues*. “*They never share the information required to complete my work. I struggled hard to get it. Later I realized that this information was already with them” (p33, male, 22 years)*.

Poor interpersonal engagement skills, lack of assertiveness, and internalizing patterns were observed in most participants. Hyper vigilant about what others think and mistrust prevent them from seeking help from others. “*I don't share. Whatever I shared in the past, turned against me. I don't even trust any, even my mother, father, siblings, or relatives.” (p44, male, 29 years)* A few felt that others wanted to see their destruction. Mr. N stated, “*someone has done something (black magic) against me... my business was good... but now nothing works in my favor.” (p6, male, 36 years)*. The themes revealed that the failure to align the stress, coping, and social support triangle disturbs the equilibrium of life. Coping with life stressors is achievable with social support. Moreover, lack of social support drains other internal and external coping resources and adds to the life stressors. Unless the person trapped in this vicious cycle of stressors gets evidence-based psychosocial support to break it, they may become vulnerable to repeated DSH, with higher fatality. The stress-vulnerability themes guide the individualized intervention content to address stressors, increase positive coping options and strengthen social support to prevent DSH in this vulnerable population.

## 4. Discussion

The study investigated risk and protective factors associated with DSH in patients presented to the emergency department of three tertiary care facilities in Kerala. Quantitative research revealed that most subjects were females, and the gender difference is attributed to the fact that women are increasingly subjected to a lot of stress related to socio-cultural roles assigned to them by people with a patriarchal mindset (36). Findings further revealed that nearly three-fourths of the study participants had a significant level of depression in the past, and all, except one, were diagnosed with a present depressive episode, which is consistent with earlier research on depression and suicidality (8–10). Suicidality, in the current study, was inversely correlated with help-seeking behavior and positively correlated with somatic symptoms, depression, and anxiety which agreed to findings from other studies (37).

The study further showed that loss of current intimate confiding relationships, conflict in current roles, generalization of hopelessness, lack of confiding relationship with the marital partner, the experience of humiliation, rejection, entrapment, and self-abasement has their historical pathways impacting the affect regulation vulnerability. Social and interpersonal relationship stressors triggered the DSH in vulnerable younger participants, whereas the family life stressors led to vulnerabilities in the married participants. Additionally, an unsupportive family environment and lack of perceived support from the spouse and in-laws were found to be critical predictors of DSH in this latter group. This is primarily due to the patriarchal influence that reinforces the fundamental helplessness attributed to married women, who thinks that their life is at stake without the support of their husband and their household.

The transition from traditional value systems of trustworthiness, altruism, and rationality (38) to impulsivity, social alienation, and negative coping makes people increasingly susceptible to acts of suicidality. This vulnerability is characterized by an egocentric worldview that accelerates the exhaustion of their limited psychosocial resources, especially when exposed to depressogenic events. The collectivist value preferences make the participants intrinsically look up to the family for support in their life stressors, and the failure of the family to fulfill this obligation would cause severe alienation of its members. Economic factors such as financial insecurity due to debt, joblessness, or underemployment were stressors for people engaged in daily wage or low-income jobs. However, a hostile and unsupportive work environment was a determinant of DSH in early-career employees in the private sector work environments.

Despite Kerala's impressive statistics in developmental indices, the high rate of suicide (16) remains an enigma that could be due to basic inequities in educational opportunities, health care, social security, and related fields (39). Exploring the cultural issues surrounding marriage and family is particularly crucial in the Kerala context as findings largely revealed a family-related antecedent for suicide among the women participants. Unequal rights in family law with regard to inheritance, and limited access to land and non-land assets, characteristics of Kerala societies, might also have predisposed suicidal ideation among the participants. Additionally, domestic violence and demands for dowry from the husband's family are prevalent discriminatory cultural determinants of suicide in Asian societies (40, 41). Women, when exposed to these stressors would end up in self-harm behaviors or suicide, when they find no way out, as divorce and separation are still frowned upon in Indian societies (16).

Findings showed that the genetic predispositions coupled with psychosocial and economic risk factors both predict and explain the negative stress reaction of suicidal behavior in this population. Additionally, the multifaceted life stressors result in the depletion of personal resources or social supports leading to deliberate self-harm behavior, which demands a complex multi-pronged intervention to prevent future attempts. Evaluating the patient for risks can help the emergency department personnel to refer them to further psychiatric consultation and psychosocial treatments which is necessary for the safety of the patient. Developing skills in identifying the signs and symptoms quickly with optimum accuracy and evaluating the risk factors is critical to guide appropriate interventions to ensure better patient outcomes. Therefore, this high-risk population needs to be proactively engaged in long-term -customized interventions with a specific focus on empowering the families to provide continued support and strengthening their social linkages to cope with life stressors and meet their socio-economic needs. Further research is required to model the intervention protocols and standardize the practice models that can be tested in emergency departments.

### 4.1. Limitations of the study

The participants' responses may have been impacted by the topic's inherent sensitivity. Eight respondents refused to participate without stating any reason, which prevented us from gathering some potentially insightful data. Although interviewers received adequate training in collecting the data to ensure accuracy, their attributes and competency variations might have influenced the data quality. However, the results of this study broaden our understanding of participants' various experiences, which would guide future interventions.

## 5. Conclusion

The subjects who had a string of unfavorable life events and stressors, though each of which was minor, insufficient access to internal and external coping resources, and insignificant social support, resulted in DSH. The complex interplay of stressors, such as the perceived lack of emotional, economic, and social coping resources, trigger and maintain the DSH behavior in the studied population. A social support-focused, flexible, and tailor-made care package delivered by an assertive engagement specialist would be an effective suicide prevention strategy.

## Data availability statement

The raw data supporting the conclusions of this article will be made available by the authors, without undue reservation.

## Ethics statement

The studies involving human participants were reviewed and approved by Rajagiri Institutional Ethics Committee. The patients/participants provided their written informed consent to participate in this study.

## Author contributions

SD: conceptualization, methodology, funding acquisition, formal analysis, project administration, resources, supervision, and roles/writing—original draft. LS and AB: formal analysis and writing—review and editing. JV: resources and writing—review and editing. NH: writing—review and editing. LJ: conceptualization, methodology, supervision, and writing—review and editing. All authors contributed to the article and approved the submitted version.
